# Influence of latitude, host body size and host body weight on helminth species richness and abundance in two Neotropical marsupials

**DOI:** 10.1016/j.ijppaw.2025.101077

**Published:** 2025-05-06

**Authors:** Bruna Silva Cirino, Sócrates Fraga da Costa Neto, Thiago dos Santos Cardoso, Arnaldo Maldonado Júnior, Rosana Gentile

**Affiliations:** a Laboratório de Biologia e Parasitologia de Mamíferos Silvestres Reservatórios, Instituto Oswaldo Cruz, Fundação Oswaldo, Av. Brasil, 4365, 21040-360, Rio de Janeiro, RJ, Brazil; b Programa de Pós-Graduação em Biodiversidade e Saúde, Instituto Oswaldo Cruz, Fundação Oswaldo Cruz, Av. Brasil, 4365, 21040-360, Rio de Janeiro, RJ, Brazil; c Fiocruz Mata Atlântica, Fundação Oswaldo Cruz, Estrada Rodrigues Caldas, 3400, Curicica, 22713-375, Rio de Janeiro, RJ, Brazil

**Keywords:** Diversity, Ecology, Latitudinal influence, Mammals, Parasites

## Abstract

Parasite species richness can be influenced by intrinsic and extrinsic factors of their hosts, such as host body size and latitude. Although these factors have been studied for several taxa, few studies have investigated them for helminths of wild mammals. The aim of this study was to evaluate the influence of host body size, body weight and latitude on the species richness and abundance of the helminths of *Didelphis aurita* and *Didelphis albiventris.* Data on helminths and the geographic coordinates of the collection localities of the hosts were obtained from the Collection of Wild Mammal Reservoirs and the database of the Laboratory of Biology and Parasitology of Wild Mammal Reservoirs. The influences of latitude and host body size and body weight on helminth species richness (HSR) and helminth abundance were analysed using linear regressions. We evaluated the effect of latitude on helminth species composition using redundancy analysis followed by ANOVA. The HSR ranged from 1 to 9 for *D. aurita* and from 1 to 7 for *D. albiventris*. We found a positive relationship between latitude and total HSR for *D. aurita* (*p* = 0.012). The ANOVA revealed the influence of latitudinal variation on the species composition variation of helminths only for *D. aurita* (*p* = 0.001). With respect to body size, we did not find a significant relationship between this variable and HSR or abundance for either species. However, we found a positive relationship between host body weight and helminth abundance for *D. aurita* (*p* = 0.004). We conclude that the increase in latitude was an explanatory factor for the increasing HSR along infracommunities, contradicting the general pattern of increasing species with decreasing latitude observed in free-living species. Moreover, host intrinsic factors may be more relevant to endoparasite occurrence and development than latitude because they directly influence the parasite niche.

## Highlights

• Latitude influenced helminth species richness of *Didelphis aurita.*
• Host body size was not a predictor of helminth species richness.
• Host body weight was a predictor of helminth abundance for *Didelphis aurita.*
• Helminth abundance was not influenced by either latitude or host body size.

## Graphical abstract

**Figure:**
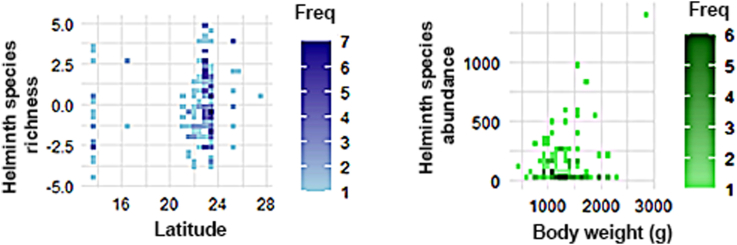

## 1 Introduction

Parasites constitute a large fraction of biodiversity; however, several questions concerning what influences parasite species richness (PSR) still need to be answered (Poulin, 2014; Morand, 2015). Parasite communities can vary across different scales of space, time and levels of organization, ranging from the infracommunity to the metacommunity (Poulin, 1997). Parasite species richness may be influenced by intrinsic factors of their hosts or extrinsic factors. For example, associations between species may be related to the overlapping areas of the geographic distributions of host species, environmental conditions, density-dependent factors and local factors (Poulin, 1997).

In relation to endoparasites, helminth species richness (HSR) may also be associated with the species-area relationship (Stronna and Fattorini, 2014). Host body size can influence parasitism because of the largest area available for parasites. Larger hosts are expected to have greater parasite richness because they have greater space and thus can harbour more species (Guégan et al., 1992; Poulin, 1997). Bordes et al. (2009) reported a positive relationship between PSR and body size in mammals. Kamiya et al. (2014) reported a positive relationship between PSR and body size and the population density in fishes and mammals. Dáttilo et al. (2020) reported a positive relationship between PSR and the body size in rodents. In contrast, Villalobos-Segura et al. (2020) reported a negative relationship between HSR and the host body mass of wild mammals, considering that more than half of this group consisted of rodent and bat hosts. In addition, larger hosts tend to be more exposed to parasite infection because they consume a greater amount of food (Poulin, 1997; Arneberg, 2002). Gregory et al. (1996) reported a positive relationship between PSR and body weight in birds and mammals. Arneberg (2002) reported a positive relationship between nematode species richness and host body weight.

Host species highly exposed to infections have a high energetic cost because of the investment in the immune response (Morand and Harvey, 2000). This investment can compromise other biological and physiological processes (*trade-offs*), such as reproduction and growth (Beldomenico and Begon, 2010; Tompkins et al., 2011). Gregory et al. (1996) suggested that host species with a high basal metabolic rate (BMR) relative to body size may favour parasite infection because they tend to have a relatively high feeding rate and daily activity. Morand and Harvey (2000) reported a positive correlation between helminth species richness and BMR.

The species richness of parasites may also be associated with factors extrinsic to their hosts. The latitudinal gradient of species richness is a general pattern in ecology for free-living species that explains the distribution and diversity of species, although there are exceptions (Willig et al., 2003). Tropical zones offer a greater range of bioclimatic factors, favouring biodiversity and environmental productivity. For marsupials, for example, there is a latitudinal gradient in marsupial species richness in the Americas (Cerezer et al., 2023) so that most of the marsupial species occur in the tropical region (Voss and Jansa, 2021) with a peak along the North and Central Andes and in part of the Atlantic Forest (Cerezer et al., 2023). For parasites, Kamiya et al. (2014) reported a weak positive association between latitude and PSR across taxa, whereas when only metazoan parasites were considered, the association observed was stronger. Preisser et al. (2022) reported a nonlinear gradient in HSR in rodents, with an increase in the tropics relative to the temperate areas, and then a further increase in HSR from the temperate areas to the poles. However, there is no study which evaluated latitudinal gradient in helminth species richness along infracommunities within a single mammal species, as the reported studies refer to HSR across different host species.

Anthropic activities have increased the interface of urban, rural and sylvatic areas (Corrêa and Passos, 2001), favouring the occurrence of generalist and synantropic species. These species become very abundant in such areas, which may promote the transmission of parasites within their populations and to other species. Opossums, for example, are abundant in urban and rural areas and have been reported as potential reservoirs of zoonotic pathogens, such as protozoa (e.g., *Leishmania infantum*, *Toxoplasma gondii* and *Trypanosoma cruzi*) (Xavier et al., 2014; Jansen et al., 2015; Bezerra-Santos et al., 2021), helminths (e.g., *Ancylostoma caninum* and *Trichinella spiralis*) (Bezerra-Santos et al., 2021), bacteria (e.g., *Chlamydia psittaci*, *Rickettsia* spp. and *Salmonella enterica*) (Bitencourt and Bezerra, 2022; Caserta et al., 2023) and viruses (e.g., *Flavivirus* and *Rotavirus*) (Bitencourt and Bezerra, 2022).

The species of opossums *Didelphis aurita* Wied-Neuwied, 1826 and *Didelphis albiventris* Lund, 1840 (Didelphimorphia, Didelphidae) have synanthropic habits, occur with high abundances in urban and rural areas and have been reported in Brazil to harbour several helminth species (Müller, 2005; Zabott et al., 2017; Costa-Neto et al., 2019; Cirino et al., 2022). Their diet may favour encounters with parasitic species. They have generalist habits and can feed on small vertebrates, invertebrates and items related to the human presence (Santori et al., 1995). In addition, these species have larger home ranges than other marsupials or small mammals, reaching more than 11 ha for *D. albiventris* and 123 ha for the congener species *D. marsupialis* (Cáceres et al., 2023), also varying according to sex and body size (Cáceres and Monteiro-Filho, 2001). Species with large home ranges have greater chances of encountering parasitic species, which can also favour parasite acquisition (Morand, 2015).

**Fig. 1:**
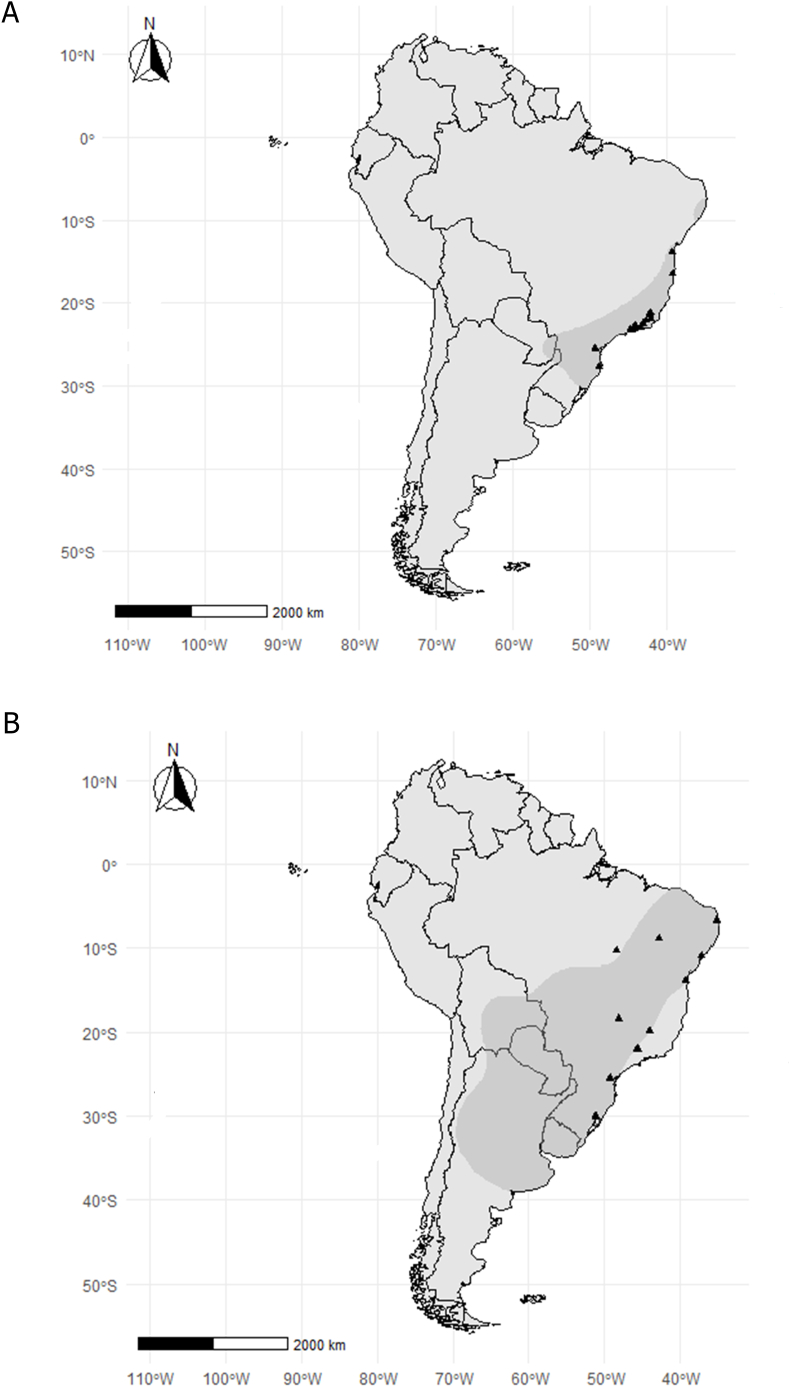
Distribution map of *Didelphis aurita* (A) and *Didelphis albiventris* (B). The points represent capture localities. The gray shade represents the known distribution area of the species, obtained in Map of Life platform (https://mol.org/).

*Didelphis aurita* occurs in the Atlantic Forest biome from the east coast of the state of Paraíba to Rio Grande do Sul in Brazil (latitude 7° S to 30° S) (Fig. 1A), with records also in the states of São Paulo, Paraná, southern Mato Grosso do Sul and Santa Catarina. In addition, the species occurs in southeastern Paraguay and northeastern Argentina (Melo and Sponchiado, 2012; Astúa et al., 2023). *Didelphis albiventris* is the opossum with the largest distribution in Brazil, occurring in the Atlantic Forest, Caatinga, Cerrado, Pantanal and Pampa Brazilian biomes. This marsupial also occurs in southeastern Bolivia, Paraguay, Uruguay, and northern and central Argentina (Melo and Sponchiado, 2012; Astúa et al., 2023). Its distribution ranges from latitude 4° S to 39° S (Fig. 1B).

In this study, we aimed to evaluate the influence of latitude, host body size and host body weight on helminth species richness, abundance and species composition in two target species, *D. aurita* and *D. albiventris,* which have wide latitudinal geographic distributions. We hypothesized that (1) helminth species richness (HSR) and abundance decrease with increasing latitude as a consequence of greater environmental productivity at lower latitudes than at higher latitudes, resulting in a loss of certain species along the latitudinal gradient towards the subtropical region because some helminth species depend on intermediate host species to complete their life-cycles, which can be absent in less productive regions; (2) larger hosts have greater HSR and abundance based on the species-area relationship; and (3) heavier hosts have greater HSR and abundance, given that an increase in body mass may be due to the ingestion of a greater variety of food items in the wild, increasing exposure to different parasites.

## 2 Materials and methods

### 2.1 Data collection

Data on parasitism by helminths and the geographic coordinates of the localities where the opossums were collected were obtained from the Collection of Wild Mammal Reservoirs (COLMASTO) and from the database of the Laboratory of Biology and Parasitology of Wild Mammal Reservoirs (LABPMR) of the Oswaldo Cruz Institute (IOC) - FIOCRUZ/RJ. Localities used in this study and the geographic range of *Didelphis aurita* are in Fig. 1A and of *D*. *albiventris* are in Fig. 1B. Geographic coordinates of each locality are in the Supplementary Material.

The animals were captured under the authorization of the Brazilian Government's Chico Mendes Institute for Biodiversity and Conservation (ICMBio, licence numbers 40869, 13373, 17131, 45839 and 26934). Capture and handling procedures were carried out in accordance with the Ethical Committee on Animal Use of the Oswaldo Cruz Foundation (CEUA licence numbers LW-39/2014 and L 36/2018) and complied with standard biosafety protocols (Lemos and D'Andrea, 2014).

Tomahawk® live traps (Hazelhurst, WI, USA) (16 × 5 × 5 inches) were used to collect the marsupials along transects with 15 capture points. Host species were identified based on external morphology, weight, body size and dental development. The animals were euthanized for helminth recovery as follows: the opossums were anaesthetized with a combination of ketamine hydrochloride (100 mg/mL) and xylazine hydrochloride (20 mg/mL) at a 1:1 ratio, administered at a dose of 0.1 mL/100 g. Intracardiac injection of 19.1 % potassium chloride was administered at a dose of 2 mL/kg when the animal was fully anaesthetized.

The recovered helminths were washed with a saline solution (0.85 % sodium chloride). Nematodes were fixed in AFA solution (93 parts 70% ethanol, 5 parts 0.4 % formalin and 2 parts 100% acetic acid) and heated to 65 °C. Some specimens were preserved in 70 % ethanol for DNA extraction. Trematodes and cestodes were compressed in cold AFA. Acanthocephalans were kept in distilled water, fixed and compressed in cold AFA for protraction of the proboscis (Amato et al., 1991).

Specimens were counted using a stereoscopic microscope and placed between a slide and a coverslip for identification under an optical microscope (Zeiss Axio Scope A1), which was coupled with an Axio Cam MRc digital camera for photomicrography. Trematodes, cestodes, and acanthocephalans were stained using Langeron's alcoholic hydrochloric carmine (Amato, 1985), dehydrated in an increasing series of alcohol, cleared and mounted in methyl salicylate.

The helminth species were identified based on morphological characteristics, as described by Vicente et al. (1997) and Anderson et al. (2009) for Nematoda, Travassos et al. (1969) for Trematoda, Gomes (1979) for Cestoda and Acanthocephala, and other relevant articles of species description.

We used data on the helminths of *D. aurita* and *D. albiventris*. A data matrix was made for each infracommunity of the target hosts, including the helminth species and the following variables: latitude of the sampled host, host body size, host body weight, host sex (male or female), helminth abundance and helminth species richness.

### 2.2 Data analysis

The presence of spatial autocorrelation in helminth species richness and abundance data was tested to investigate the independence of data among infracommunities using the Moran index (Moran, 1950). This index provides a measure of spatial dependency to detect whether a spatial pattern is clustered, dispersed, or randomly distributed in geographic space (Bivand and Wong, 2018). This index ranges from -1 (where the distribution is dispersed, with points containing distinct values that are close to each other) to +1 (where the distribution is clustered, with points containing similar values that are close to each other). Conversely, values near zero indicate no spatial autocorrelation in the data (Moran, 1950; Bivand and Wong, 2018). When spatial autocorrelation was detected, regression models were performed, and the residuals of these models were used as response variables in the subsequent analyses.

The influences of latitude, host body size and host body weight on total helminth species richness and abundance were analysed using linear regression. In this case, except for the relationships between host body weight and the variables studied, all the host specimens, including male, female, juvenile and adult hosts, were analysed. To analyse the influence of host body weight on the richness and abundance of parasites, only adult specimens were used. Lactating females were also excluded. With respect to host body size, the residuals from a regression model between body size and host sex were extracted, and these residuals were used in the final models. This was accomplished because of the sexual dimorphism in the body size of these marsupials (Cáceres et al., 2023). Finally, the effects of latitude on the helminth species composition of *D. aurita* and *D. albiventris* along infracommunities were analysed using redundancy analysis (RDA), followed by analysis of variance (ANOVA).

All calculations and analyses were performed separately for each host species. Spatial autocorrelation was investigated using the *spdep* (Bivand, 2022) and *sf* packages (Pebesma, 2018; Pebesma and Bivand, 2023). Regression and ANOVA analyses were performed using the *stats* package (R Core Team, 2024), and the RDA was conducted using the *vegan* package (Oksanen et al., 2024). All the analyses were performed in R software version 4.3.3 (R Core Team, 2024).

## 3 Results

The database contained 182 specimens of *Didelphis aurita*, 164 of which were infected with at least one helminth species. Eighty-six specimens of *D. albiventris* were recorded, of which 84 were infected. The total helminth species richness for these hosts ranged from 1 to 9 for *D. aurita* and from 1 to 7 for *D. albiventris*.

The compiled list of the helminths of *D. aurita* included 16 morphotypes of nematodes, five of trematodes, one of cestode, and one of acanthocephalan (Table 1; Supplementary Material). For *D. albiventris*, we found 12 morphotypes of nematodes, six of which were trematodes, one of which was a cestode, and two of which were acanthocephalans (Table 1; Supplementary Material).

**Table 1:** Sex (male or female), number of host specimens, body size (head-body in millimetres) and mean body weight (in grams) ± standard deviation of *Didelphis aurita* and *Didelphis albiventris* and the number of helminth species found in each phylum, Nematoda, Platyhelminthes (classes Cestoda and Trematoda) and Acanthocephala.

Host	*Didelphis aurita*			*Didelphis albiventris*	
Sex (N)	Males (n = 107)	Females (n = 72)		Males (n = 50)	Females (n = 34)
Body size (mm)	364.66 ± 63.19	367.94 ± 70.09		326.73 ± 66.73	326.35 ± 70.55
Body weight (g)	1124.86 ± 481.29	1026.87 ± 394.12		791.33 ± 484.17	667.65 ± 386.19
Nematoda	16			12	
Trematoda	5			6	
Cestoda	1			1	
Acanthocephala	1			2	

**Fig. 2:**
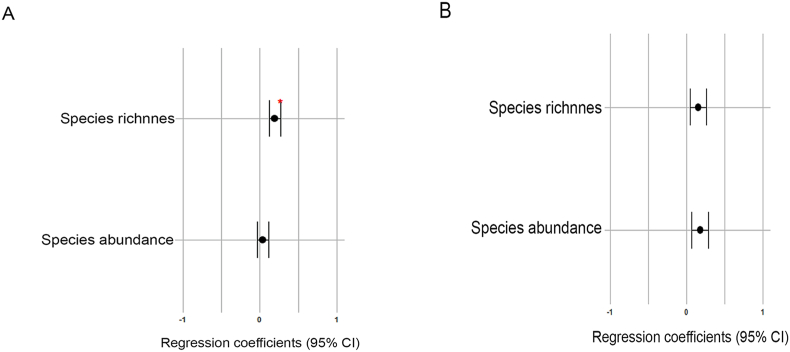
Forest plot showing the influence of latitude on helminth species richness and abundance in *Didelphis aurita* (A) and *Didelphis albiventris* (B) using linear regression. The width of the lines represents the 95 % confidence interval. Red asterisk indicate significant pairwise differences at the α level of 0.05 (∗). The x axes were standardized within the -1 and +1 ranges, and the coefficient values were adjusted for these ranges for better visualization of the plots.

**Fig. 3:**
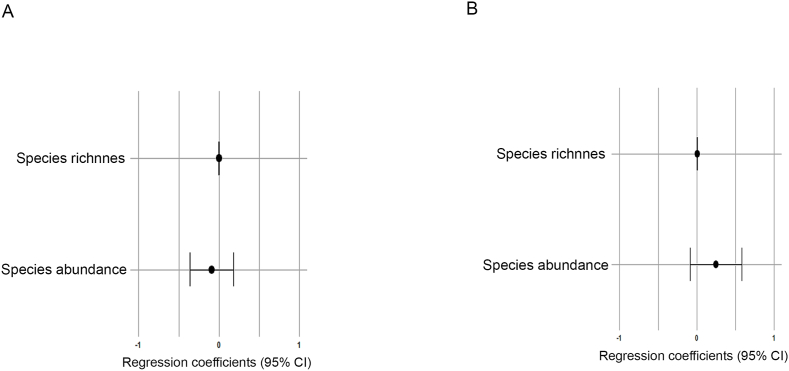
Forest plot showing the influence of host body size on helminth species richness and abundance in *Didelphis aurita* (A) and *Didelphis albiventris* (B) using linear regression. The width of the lines represents the 95 % confidence interval. No significant pairwise difference at the α level of 0.05 was found. The x axes were standardized within the -1 and +1 ranges, and the coefficient values were adjusted for these ranges for better visualization of the plots.

**Fig. 4:**
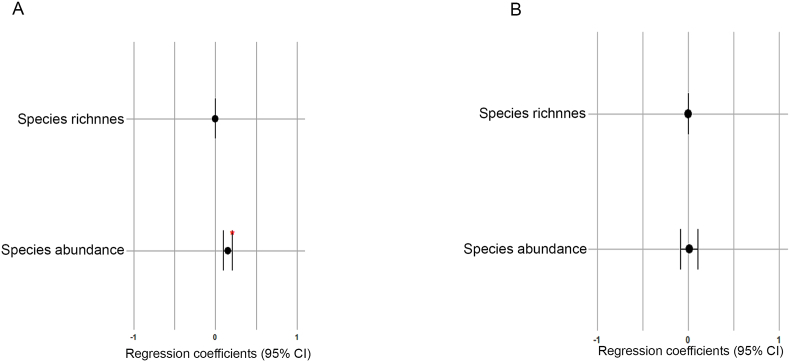
Forest plot showing the influence of adult host body weight on helminth species richness and abundance in *Didelphis aurita* (A) and *Didelphis albiventris* (B) using linear regression. The width of the lines represents the 95 % confidence interval. Red asterisk indicate significant pairwise differences at the α level of 0.05 (∗). The x axes were standardized within the −1 and +1 ranges, and the coefficient values were adjusted for these ranges for better visualization of the plots.

Spatial autocorrelation was observed in nine of the 12 comparisons (Table 2). In those cases, except for the relationships between body size and helminth abundance in *D. aurita* and *D. albiventris* and between latitude and abundance in *D. albiventris* (Table 2), the residuals of the autocorrelations were used in the regression analyses. We found a significant relationship between latitude and species composition (*p* = 0.001; Table 3) and a significant direct relationship between latitude and total species richness for *D. aurita* (*p* = 0.012; Table 4; Fig. 2A), indicating that the HSR was greater at higher latitudes. These relationships were not found for *D. albiventris* (Tables 3 and 4, Fig. 2B). With respect to host body size, we found no significant relationship between this variable and HSR or abundance for either species (Table 5; Fig. 3). However, a significant direct relationship was found between host body weight and helminth abundance for *D. aurita* (*p* = 0.004; Table 5; Fig. 4).

**Table 2:** Values of the spatial autocorrelation analyses according with the Moran Index for each parameter used in the regression analyses of helminths species richness and abundance for *Didelphis aurita* and *Didelphis albiventris*. SD – standard deviation; *p* value – probability; ∗ – significant results.

Parameter	Moran's I	SD	*p* value
*Didelphis aurita*			
Species Richness x Latitude	0.45	4.84	6.49 × 10−7∗
Abundance x Latitude	0.23	2.76	0.002∗

Species Richness x Body size	0.47	4.82	7.11 × 10−7∗
Abundance x Body size	0.12	1.39	0.082
			
Species Richness x Body weight	0.6	4.91	4.63 × 10−7∗
Abundance x Body weight	0.28	2.47	0.007∗
			
			
*Didelphis albiventris*			
Species Richness x Latitude	0.3	2.3	0.011∗
Abundance x Latitude	0.06	0.54	0.292
			
Species Richness x Body size	0.43	3.17	0.001∗
Abundance x Body size	0.14	1.13	0.129
			
Species Richness x Body weight	0.69	3.4	3.331 × 10−4∗
Abundance x Body weight	0.35	1.77	0.037∗

**Table 3:** Results of the analysis of variance (ANOVA) for the effect of latitude on helminth species composition for *Didelphis aurita* and *D. albiventris* along the infracommunities. F – analysis of variance test; DF – degrees of freedom; *p* value – probability; ∗ – significant results.

Hosts	F	DF	*p* value
*Didelphis aurita*	4.679	180	0.001∗
*Didelphis albiventris*	1.439	84	0.149

**Table 4:** Results of the linear regressions between latitude and total helminth species richness and helminth abundance for *Didelphis aurita* and *Didelphis albiventris*. Beta – regression coefficient; F – analysis of variance test, DF – degrees of freedom; R^2^ – coefficient of determination; *p* value – probability; ∗ – significant results.

Parameter	Beta	F	DF	R^2^	*p* value
*Didelphis aurita*					
Richness	0.128	6.374	180	0.028	0.012∗
Abundance	2.072	0.135	180	-0.004	0.713

*Didelphis albiventris*					
Richness	0.024	1.811	84	0.009	0.182
Abundance	3.549	2.346	84	0.016	0.129

**Table 5:** Results of the linear regressions between body size and body weight (only for adult specimens) with helminth species richness and abundance for *Didelphis aurita* and *Didelphis albiventris*. Beta – regression coefficient; F – analysis of variance test, DF – degrees of freedom; R^2^ – coefficient of determination; *p* value – probability; ∗ – significant results.

Parameters	Beta	F	DF	R^2^	*p* value
*Didelphis aurita*					
Richness x body size	-1.05 × 10^−3^	0.192	168	-0.004	0.661
Abundance x body size	-0.105	0.143	168	-0.005	0.708

Richness x body weight	-3.62 × 10^−4^	0.584	105	-0.004	0.343
Abundance x body weight	0.145	8.703	105	0.068	0.004∗


*Didelphis albiventris*					
Richness x body size	0.005	3.708	81	0.032	0.058
Abundance x body size	0.267	0.618	81	-0.005	0.434

Richness x body weight	2.97 × 10^−4^	0.284	35	-0.019	0.597
Abundance x body weight	-0.010	0.012	35	-0.027	0.912

## 4 Discussion

Some patterns of species richness are observed in several taxonomic groups. The relationship between latitudinal gradients and biodiversity has been shown for several free-living taxa (Willig et al., 2003). Some studies have also reported this relationship in species with a parasitic lifestyle (Poulin, 1995; Poulin and Morand, 2000; Kamiya et al., 2014; Morand, 2015; Dáttilo et al., 2020; Villalobos-Segura et al., 2020). Nonetheless, several studies have reported that parasite species richness is unrelated to latitude or exhibits a latitudinal gradient in species richness that differs from the pattern observed in free-living species; that is, richness increases with increasing latitude (Poulin, 1995; Lindenfors et al., 2007; Kamiya et al., 2014; Torchin et al., 2015; Preisser, 2019; Johnson and Haas, 2021).

This is the first study to analyse the effects of latitude on the species richness and abundance of helminths within Neotropical marsupial host species. There was an increase in total helminth species richness with increasing latitude in *D. aurita*, which does not support the first hypothesis for this host. This result indicates a greater number of helminth species per infracommunity with increasing distance from the host to the equator, although this relationship was not found for helminth abundance. The same relationships found in the present study have already been reported in a few studies that investigated this pattern of HSR in mammals. Lindenfors et al. (2007) reported a positive relationship between latitude and HSR in carnivores. Kamiya et al. (2014) reported a positive, albeit weak and nonsignificant, relationship between latitude and parasite species richness across parasite taxa; however, when only metazoan parasites were considered, the association was significant and positive. Preisser et al. (2022) reported a significant but nonlinear gradient between latitude and HSR in rodents, which was greater at lower latitudes, decreased at temperate latitudes and increased again at polar latitudes.

However, latitude does not seem to be a universal factor and is strongly influenced by sampling (Torchin et al., 2015). Some studies with helminths have revealed changes in the results when analysed at different scales and between different taxonomic levels (Preisser, 2019; Preisser et al., 2022). This can be attributed to parasite‒host coevolution, resulting in differences in the response and adaptation of each helminth species (Combes, 2001; Preisser, 2019).

Considering the geographical distribution of the analysed host species, the latitudinal extent of the sampling was highly representative for the species *D. albiventris*. Although it was not equally representative for *D. aurita*, the data obtained still yielded significant results for the HSR. Notably, the distribution of these hosts is almost entirely in the tropical zone, and the study analysed variations between infracommunities within each species.

The second hypothesis was not supported by the present study, as we did not find a relationship between host body size and helminth species richness or abundance. However, among the host-intrinsic factors related to parasite species richness, body size can be considered one of the most important because it is directly associated with the species-area relationship. Bordes et al. (2009) reported a positive relationship between the body size of mammals and the species richness of parasites. Kamiya et al. (2014) reported a positive relationship between fish and mammals. Dáttilo et al. (2020) reported a positive relationship for rodents. Cirino et al. (2020) reported a significant and positive relationship between body size and the abundance of helminths of *Metachirus myosuros* and between body size and HSR when only female hosts were analysed. Cirino et al. (2022) reported a positive relationship between body size and the total abundance of the most prevalent helminths of *D. albiventris*. On the other hand, Villalobos-Segura et al. (2020) reported the opposite relationship between HSR and the body mass of wild mammals, with most hosts being rodents or bats. These results indicate that this relationship between host body size and the richness or abundance of helminth parasites seems to be more evident among taxa of different host species than within a host species, because there is a larger variation in body size among host species than among individuals within a certain species.

The relationship between host body weight and helminth abundance corroborates part of the third hypothesis. Gregory et al. (1996) reported a positive relationship between body weight and the PSR in birds and mammals. Arneberg (2002) reported a positive relationship between host body weight and nematode species. A better-nourished host may have a more efficient immune system to prevent colonization by different helminth species but may favour an increase in the abundance of some already established species (Combes, 2001). The results of the present study regarding the positive influence of body weight on helminth abundance in *D. aurita* corroborates this idea. In addition, helminths compete for space and resources within the host. The abundance of a parasite species may be the result of interspecific competition, where a better-established species may prevent the colonization of other species (Combes, 2001).

This study also contributed to the increase in the list of host species and geographic distribution of two helminth species, which are new occurrence records for these hosts. *Travassostrongylus sextus* Freitas (1937) (Rhabditida, Viannaiidae) was found in *D. aurita* in Igrapiúna, Bahia, and in *D. albiventris* in Curitiba, Paraná. *Travassostrongylus tertius* Travassos et al., 1969 (Rhabditida, Viannaiidae) was found in *D. aurita* in Paraty, Rio de Janeiro. These helminth species were reported for the marsupial species *Metachirus myosuros* (Temminck, 1824) by Freitas (1937). All the other helminth species identified in this study have previously been reported as parasites of *Didelphis aurita* and *D. albiventris*. The occurrence of a given parasite on a given host species can be attributed to geographical factors due to the parasite sharing and spillover, and to taxonomic factors due to the host-parasite coevolution (Poulin, 2007).

## 5 Conclusions

It is important to note that latitude is a proxy for several factors that vary in geographic space, such as variations in temperature, humidity, rainfall, and habitat availability (Johnson and Haas, 2021). This fact may have contributed to the relationship between latitude and the helminth species richness of *D. aurita*. This effect, however, was considered low. Other ecological factors not analysed here may also be related to HSR, such as diet variations depending on resource availability, behaviour, host population size and host home range size (Kamiya et al., 2014; Morand, 2015). In addition, the PSR can also be influenced by geographical and immunogenetic factors between hosts (Morand, 2015; Dallas et al., 2020). The low influence of latitude on the variation in helminth species composition and the positive influence of host body weight on the helminth abundance observed for *D. aurita* suggest that although extrinsic and intrinsic factors determine the occurrence and abundance of parasites in wild animals, host intrinsic factors may be more relevant to endoparasite occurrence and development because they have a direct and closer influence on the niches of these parasites.

## CRediT authorship contribution statement

**Bruna Silva Cirino:** Writing – review & editing, Investigation, Formal analysis, Data curation. **Sócrates Fraga da Costa Neto:** Writing – review & editing, Investigation, Data curation. **Thiago dos Santos Cardoso:** Writing – review & editing, Investigation, Formal analysis. **Arnaldo Maldonado Júnior:** Writing – review & editing. **Rosana Gentile:** Writing – review & editing, Supervision, Investigation, Funding acquisition, Formal analysis, Conceptualization.

## Funding

This work was supported by the Conselho Nacional de Desenvolvimento Científico e Tecnológico – CNPq – PPBio Rede BioM. A (457524/2012-0), Fundação Carlos Chagas Filho de Amparo à Pesquisa do Estado do Rio de Janeiro - FAPERJ (RG grant number E−26/010.001597/2019), Instituto Oswaldo Cruz (IOC – FIOCRUZ), Serviço de Referência em Saúde at Fundação Oswaldo Cruz and Programa de Pós-Graduação em Biodiversidade e Saúde (IOC - FIOCRUZ). B.S.C. received grants from Instituto Oswaldo Cruz and from Coordenação de Aperfeiçoamento de Pessoal de Nível Superior (CAPES), Brazil, finance code 001. TSC received grants from Fundação Carlos Chagas Filho de Amparo à Pesquisa do Estado do Rio de Janeiro - FAPERJ, process number E−26/204.420/2021. RG received a researcher fellowship from CNPq [303643/2022-6].

## Declarations of interest

None.
