# Supplementary material for: Influence of latitude, host body size and host body weight on helminth species richness and abundance in two Neotropical marsupials
Source: Int J Parasitol Parasites Wildl. 2025 May 6;27:101077. doi: 10.1016/j.ijppaw.2025.101077 (PMC12152593; doi:10.1016/j.ijppaw.2025.101077)
Supplement: Multimedia component 1 [file mmc1.docx]

**Influence of latitude, host body size and host body weight on helminth species richness and abundance in two Neotropical marsupials**

Bruna Silva Cirino ª^,b^, Sócrates Fraga da Costa Neto ^c^, Thiago dos Santos Cardoso ª^,*^, Arnaldo Maldonado Júnior ª, Rosana Gentile ª

ª Laboratório de Biologia e Parasitologia de Mamíferos Silvestres Reservatórios, Instituto Oswaldo Cruz, Fundação Oswaldo, Av. Brasil, 4365, 21040-360, Rio de Janeiro, RJ, Brazil

^b^ Programa de Pós-Graduação em Biodiversidade e Saúde, Instituto Oswaldo Cruz, Fundação Oswaldo Cruz,
Av. Brasil, 4365, 21040-360, Rio de Janeiro, RJ, Brazil

^c^ Fiocruz Mata Atlântica, Fundação Oswaldo Cruz, Estrada Rodrigues Caldas, 3400, 22713-375, Curicica, Rio de Janeiro, RJ, Brazil

* Corresponding author.

E-mail addresses: brunascirino@gmail.com (B.S. Cirino), socratesfneto@yahoo.com.br (S.F. Costa-Neto),*

*thgo_cardoso@yahoo.com.br (T.S. Cardoso), maldonad@ioc.fiocruz.br (A. Maldonado-Júnior), rgentile@ioc.fiocruz.br (R. Gentile)

**Legend**

**Supplementary Material.** Helminth species found in *Didelphis aurita* and *Didelphis albiventris* including type of life-cycle, locality, geographical coordinate in decimal degrees (DD) (datum WGS84), number of infected hosts per helminth species, mean abundance with standard deviation (SD), and prevalence (%) with 95% confidence limits of infection for each locality.
